# Replacing cow’s milk with plant-based drinks: consequences for nutrient intake of young children on a balanced diet in Germany

**DOI:** 10.1186/s41043-025-00836-z

**Published:** 2025-03-28

**Authors:** Mathilde Kersting, Hermann Kalhoff, Katja Zahn, Aziza Janice Belgardt, Kathrin Sinningen, Thomas Lücke

**Affiliations:** 1 Research Department of Child Nutrition, University Hospital of Pediatrics and Adolescent Medicine, St. Josef- Hospital, Ruhr-University Bochum, Bochum, Germany; 2 Pediatric Clinic, Klinikum Dortmund, Beurhausstrasse 40, D-44137 Dortmund, Germany

**Keywords:** Nutrition, Sustainability, Plant-based drinks, Micronutrient supply, Young children

## Abstract

**Purpose:**

The transition to a planetary health diet goes along with the increase of plant-based alternatives to milk available on the market. Therefore, it is necessary to assess the consequences of replacing milk with plant-based drinks on the nutrient intake of young children on a balanced diet.

**Methods:**

An internet search was conducted on plant-based drinks available on the German market. Scenarios of nutrient intake were calculated in which fluid cow’s milk was replaced by plant-based drinks in the menus of the Optimized Mixed Diet (OMD), the guideline balanced diet for children in Germany.

**Results:**

Six different drinks made from legumes (soy), cereals (oats) and nuts (almonds) in three characteristic product types (basic, no fortification / basic + fortification / special products for children) were analyzed. The replacement had hardly any effect on the intake of energy and protein. However, the consequences for micronutrients were remarkable. By replacing milk with non-fortified plant-based drinks (around 80% of products on the market) the daily intake of calcium, vitamin B2, B12, and iodine was reduced to around 50%; with the fortified products, only the intake of iodine was reduced. With the children’s products, the supply of the micronutrients examined was maintained within the OMD.

**Conclusions:**

The lack in important nutritional components (calcium, B12, B2, and iodine) as a consequence of replacement of cow’s milk with most of the plant-based drinks on the current market on nutrient intake of young children can hardly be foreseen by parents. Even minor-looking changes to a balanced diet require an expert opinion of advisors.

**Trial registration:**

Number and date of registration for prospectively registered trials.

## Background

In children’s nutrition in western countries like Germany cow’s milk (in the following: ‘milk’) is a traditional component. Milk is an important source of nutrients such as high-quality protein, minerals, especially calcium and zinc with high bioavailability, and vitamins, especially B12 and B2. Moreover, milk provides various bioactive components that support growth and developmental functions [1].

Recently, a global paradigm shift in dietary habits has been proposed considering planetary health and sustainability in addition to nutritional requirements. In typical Western diets, this means an increase in plant-based foods at the expense of animal-based foods. This has been advocated for the whole population, including children from the age of 2 years [2]. As part of the transition towards a planetary health diet, plant-based milk alternatives increasingly conquer the market. In Germany, a large segment are plant-based drinks from various sources used as substitute to fluid milk [3]. Current sales data confirm the increasing use of plant-based drinks along with decreasing sales of milk [4]. As a consequence, existing dietary guidelines for children need to be reviewed according to what extent dairy products can be replaced with plant-based alternatives without compromising nutrient supply. This relates especially to groups with special dietary needs like infants and young children [5].

In the course of early childhood, special infant nutrition continues to develop to the mixed family diet with common foods, including milk and dairy products. Accordingly, in the German guidelines for child nutrition, the so-called ‘Dietary Schedule for the first year of life’ [6] seamlessly transitions to the concept of the Optimized Mixed Diet (OMD) which covers all age-groups between 1 and 18 years [7]. The present assessment focuses on the OMD and the age group of young children aged 1–3 years.

*Objective* was to build scenarios of the OMD in which fluid milk was replaced by various types of plant-based drinks available on the market in Germany as well as to assess the consequences of the replacement on nutrient intake. The results are intended to inform dietary counselling about the newly emerging prospects (either risks or advantages) of changing the traditional part of milk in the diet of young children.

## Methods

### Market observation for plant-based drinks

The products used for the scenarios had to meet the following criteria:

- representing the plant groups legumes, nuts, and cereals to mirror the main product segments of plant-based drinks in Germany.
- including plant-based drinks intended for infants and young children.
- providing comprehensive information on the specific product composition.

As a first step, a product search was carried out on the internet (May 2023) where information on ingredients and nutrient content was available for the product. As a second step, additionally, information was requested from the manufacturers if necessary, e.g. to ensure completeness of fortified nutrients. The products with the most comprehensive labelled information on nutrient content in the respective product group were used for the scenarios.

### Scenarios of the OMD

The OMD is based on a 7-day menu that considers customary food habits of German families, with 5 meals per day (3 main meals, 2 snacks). Common nutrient-dense foods and home cooking of meals are preferred, highly processed foods are accepted occasionally. The nutrient densities (g (mg)/1000 kcal) of the 7-day menu ensure that age groups from 1 to 18 years achieve the German reference values of macro- and micronutrients provided that the age-appropriate energy requirement is covered [7].

In the original 7-day menu of the OMD, the foods in the food group ‘dairy products’ can be differentiated into separate subgroups. This shows that dairy in the OMD consists mainly of fluid milk, followed by much smaller amounts of yoghurt and even smaller of cheese and milk fats (Table 1). The portion of fluid milk [219 g/d] has been replaced by the different plant-based drinks in the scenarios.

**Table 1:** Daily amounts of milk and other dairy products for the age group 1-3yrs in the OMD

Spalte1	Amount (g)	Energy (kcal)	Energy (% OMD)
Milk, fluid	219.0	105.3	7.8
Yoghurt	53.6	30.0	2.2
Cheese	16.4	41.7	3.1
Cream & Butter	3.1	5.9	0.4

For the scenarios, the amount of fluid milk (drinking, cooking) in the 7-day menu of the OMD for the age-group 1–3 years was replaced by the selected plant-based alternatives, all other food and drink remained. For the nutritional assessment, nutrients that are typical for dairy products (protein, calcium, Vitamins B2, B12) or are especially important for young children (iron, iodine) were chosen. The daily intake of energy and the selected nutrients with the modified 7-day menus was compared with the original OMD (with milk) as reference. To calculate energy and nutrients intake, a common software (DIÄT-2020 Soft & Hard, D. Beyer, Rimbach, Germany) was used. Nutrient values including cow’s milk were obtained from the German Food Code and Nutrient Data Base (Bundeslebensmittelschlüssel, BLS, Version II.3) [8] that has been used repeatedly in international studies in European adolescents [9].

## Results

### Composition of plant-based drinks

**Fig. 1:**
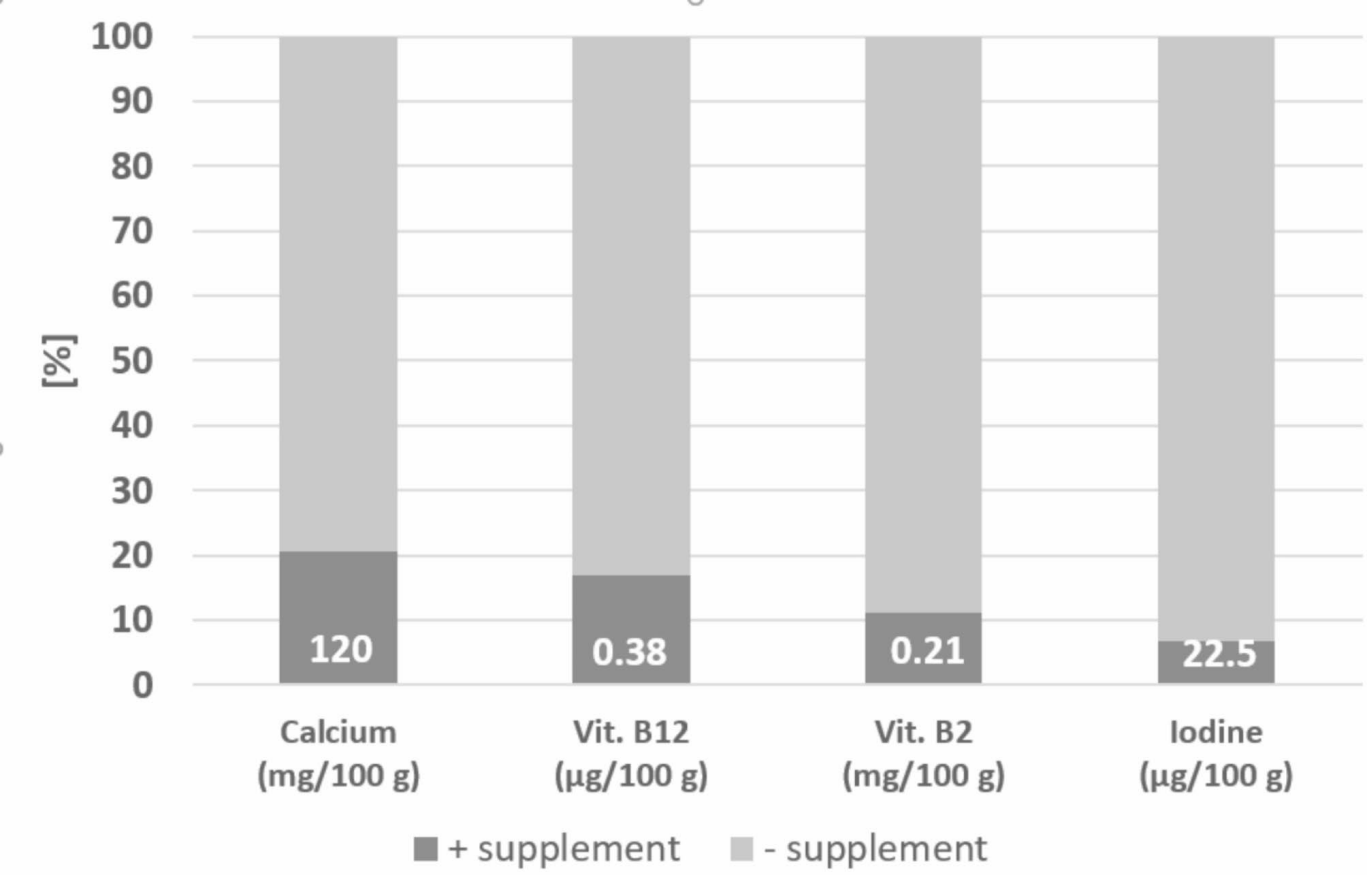
Percentage of plant drinks in a market survey fortified with calcium, Vitamin B12, Vitamine B2, and Iodine (according to data given in [10]). The uniform concentration of nutrients in supplemented drinks, identical with cow’s milk is indicated in the columns (except 2/6/0/5 products for Ca/Vitamine B12, Vitamine B2/ Iodine)

In the latest market survey (2024) by a large consumer organization in Germany, which covered a total of 160 different drinks and was focused on nutrient fortification. This total supply of drinks was distributed among the plant groups cereals (66.9%), nuts (x 17.5%), and legumes (15.6%). The prevalence and amount of fortified micronutrients (calcium, Vit B12, Vit B2, iodine) is summarized in Fig. 1.

Two important findings for the present work become apparent: (1) fortification is rare overall with a maximum of 20% of products for calcium and less than 10% for iodine, (2) fortified amounts correspond almost uniformly to the content in cow’s milk (8).

For this analysis, six different drinks were considered representing the three main food groups legumes (by soy), cereals (by oats) and nuts (by almonds) as well as three characteristic product categories (basic ingredients, no fortification / basic ingredients + fortification / special products intended for children). The two products intended for children were a so-called ‘growing up’ drink for young children (1–3 yrs.) and a special dietetic product (formula) for infants and young children with cow’s milk intolerance, both products based on soy.

**Fig. 2:**
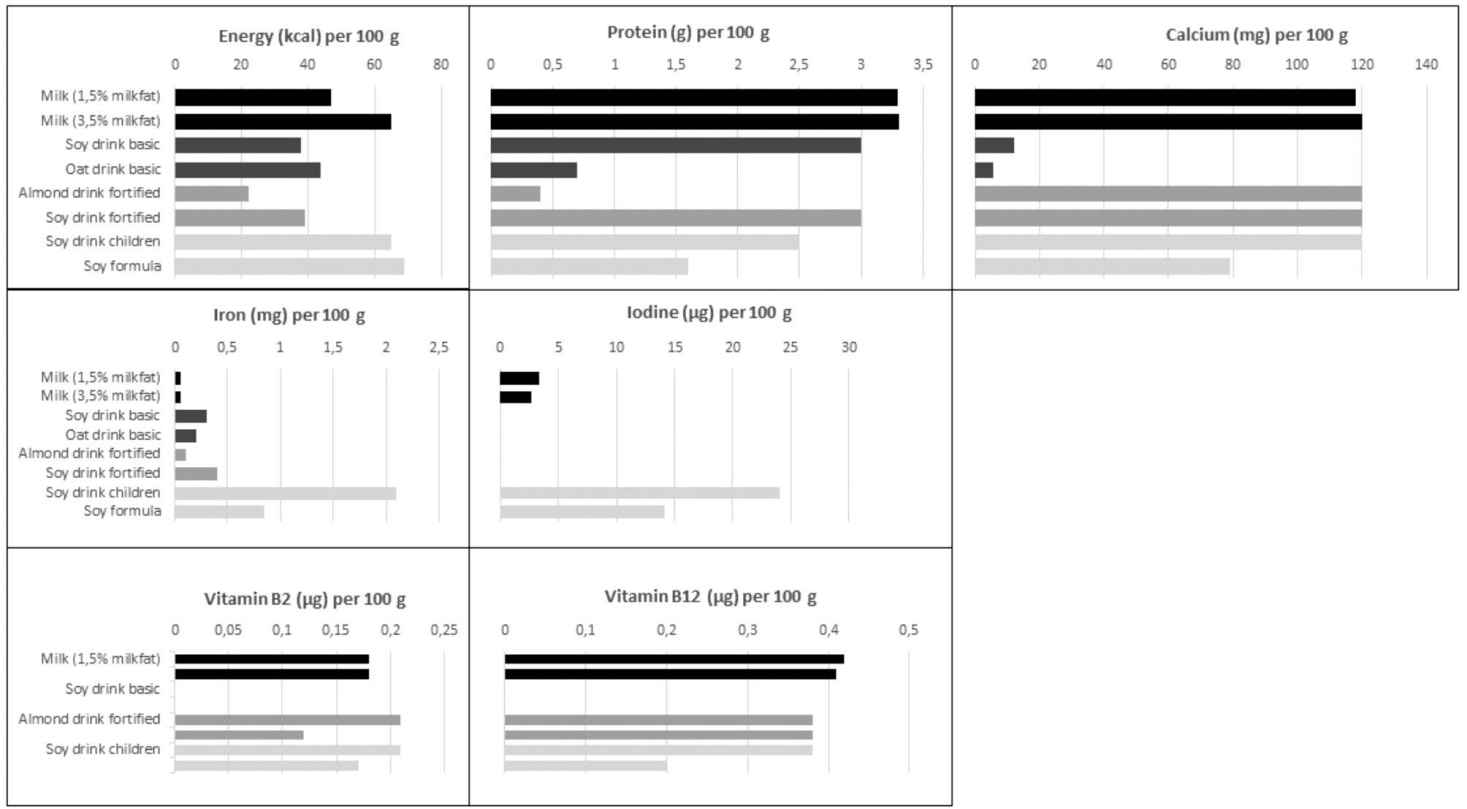
Content of energy and nutrients in selected plant-based drinks compared to milk

The composition of the drinks is displayed in Fig. 2, with milk with 1.5% fat (standard in the OMD) and milk with 3.5% fat for comparison.

The drinks differ greatly in their content of energy and nutrients compared to each other and compared to milk. With the exception of the low-energy almond drink, the energy content of the four common drinks is similar to that of low-fat milk, while the energy content of the two children’s products is similar to that of whole milk. Compared to milk, the oat and almond drinks are significantly lower in protein, while the soy drinks come close to milk, with the exception of a lower protein content of the dietetic soy formula for infants and young children.

With regard to micronutrients, the two fortified products (soy, almond) and the growing-up drink come close to milk for calcium and partly for Vitamin B2 and B12, while the soy formula is enriched to a lower extent. In the non-fortified drinks (soy, oat) the micronutrients considered here are hardly present. The two children‘s products stand out due to their multiple fortification, including iodine and iron, that do not otherwise occur in the drinks.

### Scenarios of the OMD

**Fig. 3:**
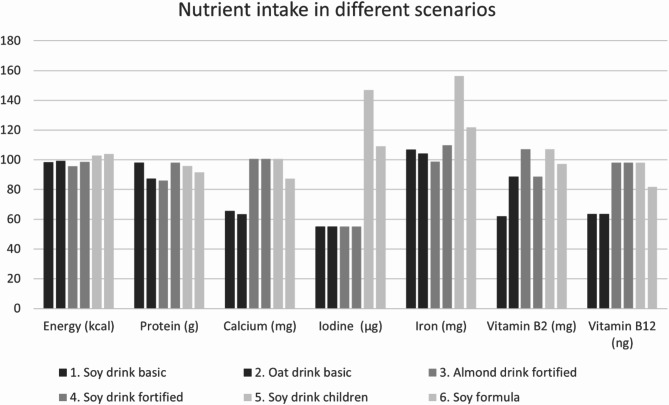
Scenarios of energy and nutrient intake when replacing fluid milk by different plant-based drinks (% of the original OMD) (Columns always from 1 on the left to 6 on the right)

The results of the scenarios of the OMD are shown in Fig. 3. While the intake of energy and protein with the OMD is hardly affected by the replacement of milk, the consequences for micronutrients differ characteristically between the nutrients and the drinks.

With the basic non-fortified products, the daily intake of calcium, iodine and the vitamins B2 and B12 is only about half the intake with the original OMD. In contrast, when using the fortified products, the nutrient-supply of the original OMD is almost achieved, with the exception of iodine, which was not fortified in the selected drinks. With the children’s products, however, the supply of all considered micronutrients of the OMD can be achieved or even significantly increased in the case of iodine and iron, the latter especially with the growing-up drink.

## Discussion

### Overall

In wealthy countries, the trend towards reducing animal-based foods in favor of plant-based alternatives has led to a need to review established dietary guidelines and to determine whether the balanced nutrient intake may be significantly compromised by these changes. Using the OMD as a model for feeding of young children, the specific scenarios of replacing liquid milk with plant-based drinks showed that:

1. The replacement of milk leads to a small reduction of less than 10% of energy intake, but the intake of important micronutrients from milk is significantly reduced and may become critically low.
2. The consequences for nutrient intake depend less on the kind of plant used as substitute and much more on the nutrient fortification by the manufacturers.

### Products and nutrients

Plant-based drinks are increasingly discussed in the literature, with the focus mostly on the product level, e.g., on differences to the composition of milk or differences in composition between drinks depending on the base plant [11–13]; whereas papers discussing the impact of plant-based drinks on the diets of children [14] are rare. Milk is a key source of iodine in many countries [15], but several studies have shown that most plant-based milk alternative drinks on the market are not fortified with iodine [15–17]. As these unfortified drinks have a low concentration of iodine, consumers of unfortified milk alternatives may be at risk for a lower iodine intake and status than cow’s milk consumers [18].

The drinks examined here reflect the range of products marketed in Germany [3] quite well. A special feature of our survey is the additional evaluation of products that are offered especially for young children. This proved to be worthwhile because of the fundamental differences in the composition of these children’s products and the products for general consumption.

The soy-based formula belongs to the category of Foods for Special Medical Purposes (FSMP) defined by EU law and complies with the EU nutrient regulations for infant and follow-up formula [19]. The growing-up soy-based product is closer to milk than the formula, but the European Food Safety Authority (EFSA) stated that special young child formula cannot *be considered as a necessity to satisfy the nutritional requirements of young children*. EFSA considers fortified young-child formula, as one of several means to increase n-3 PUFA, iron, vitamin D and iodine intakes in infants and young children living in Europe with inadequate or at risk of inadequate status of these nutrients [20].

Depending on the base plant there are well-known differences in protein: in soy, protein content and biological value are higher than in cereals and nuts. In a balanced mixed diet for young children with a moderate proportion of animal products, as in the OMD, the protein quality of the milk substitute is generally not critical due to the high protein supply. However, soy-based drinks could be advantageous over other plants groups if protein intake is scarce, for example in restrictive diets for young children.

On the other hand, soy is not uncontroversial in infant and young-child nutrition. Currently, in the EU, most infant and follow-up formula are based on cow’s milk, isolated soy protein is permitted as well (with a slightly higher protein content to compensate for the lower protein quality). In the view of pediatric nutritional medicine, the use of soy products for healthy infants should be limited to a few medical indications (galacatosemia, congenital lactose intolerance) or explicit parental preferences (desire for vegan nutrition or other ideological reasons) [21]. In these cases, medical supervision and appropriate supplementation are recommended.

The dietary assessment of plant-based drinks must also take into account the bioavailability of the nutrients, which may be lower than in milk, e.g., in the case of calcium, depending on the calcium salt and the food matrix [22, 23]. The fortification of the examined drinks for general consumption largely mimics the contents in milk, whilst the products intended for infants and young children additionally include the critical nutrients iron and iodine.

The non-fortified drinks in our study which reflect about 80% of the products on the market in Germany are often so-called “Organic products”. For this product type, fortification is made more difficult by EU Regulation 2018/848 [24], originally with the aim of protecting these products from (undesirable) additives or impurities. However, parents in Germany seem to perceive the label “organic” as associated with health benefits for their children [25]. It may be a challenge to convincingly inform parents that organic products may also be disadvantageous for their children’s nutrient supply in individual cases.

### Consequences for nutrient supply

Here, the replacement of milk with plant-based drinks was investigated with a focus on the consequences for a guideline diet, which is designed to be nutritionally balanced and safe. The model of the OMD facilitated such an approach, as this concept is based on a detailed 7-day menu and thus can show the specific consequences of replacing individual foods for the resulting nutrient intake. The scenarios were designed to be realistic and low-threshold, by only replacing the fluid milk in the menu 1:1 with plant-based drinks; the preparation of the meals otherwise remained unchanged. As the solid dairy products (yoghurt, cheese) were retained, which make up around a third of the OMD dairy group in terms of consumption and around half in terms of energy intake, at first glance one would expect rather minor effects on nutrient intake.

Indeed, the protein quantity and quality of the OMD is hardly affected by replacing milk with plant-based drinks. With restrictive dietary patterns, however, plant-based drinks with low protein quality could impair the protein supply in periods with high protein requirements due to intensive growth.

In case of micronutrients, the scenarios show distinct negative consequences for intake when using plant-based drinks without fortification. This applies in particular to calcium and vitamin B12, as these two micronutrients just reach the German reference intakes in the original OMD [7]. The deficiencies with plant-based drinks are difficult to replenish with other natural common foods. With drinks, fortified corresponding to milk, the safety of the nutrient intake of the OMD for young children is maintained.

A special issue is the critical nutrient iodine in children. As in other countries without universal salt-iodization policy, the supply in Germany is inadequately low [26] and even trending downwards [27–29]. In addition to iodized table salt, the main source of iodine for children is milk. Many young children do not yet participate fully in the family diet and cannot yet benefit fully from the use of iodized salt. Iodine fortification of plant-based drinks would be beneficial for iodine supply of young children. Thus, manufacturers of plant-based alternatives (such as plant-based milk alternatives) should be encouraged to fortify products to be equivalent to animal-based products (for example, cows’ milk).

Unlike iodine, cow’s milk is an insignificant source of iron. With the original OMD, iron intake and sufficient bioavailability is ensured from the mixture of animal-based and plant-based foods. Accordingly, our scenarios show that the iron intake in the OMD is not compromised by the replacement of milk with plant-based drinks.

However, if plant-based drinks are designed with a significantly higher iron content than milk, then the iron intake increases above the OMD amount, however, the selection of suitable iron compounds is a prerequisite for sufficient bioavailability. High iron intake in young children may be of interest in certain individual cases (according to medical indication).

In the actual diet of young children in Germany, milk consumption has been reported to be close to the recommendation, and calcium intake almost in line with the nutrient reference [30, 31]. The scenarios of this paper therefore appear to be transferrable to the actual diet of young children in Germany.

### Child-friendly compromise of healthy and sustainable diet

In principle, the OMD is a ‘modern’ plant-based mixed diet, based on three simple rules for food selection which are to consume beverages and plant-based foods generously, animal-based foods moderately and high-fat, high-sugar foods sparingly [7]. If such a diet is to achieve a further gain for the environment through modifications, then this possible gain should be reasonably quantifiable. At present, reliable sustainability data for “plant-based substitutes for animal foods” are not generally available [32].

The safety of established guideline diets is based not only on science but also on observations of children thriving on these diets. With the increasing introduction of newly designed substitute products for animal foods, these safety aspects would have to be adequately demonstrated for the individual new product groups [33].

### Strengths and limitations

Among the strengths of this analysis is the exact data of consumption of individual foods in the OMD 7-day menu, even with a specification of fluid milk (within the dairy group) that enables realistic exchange scenarios.

The use of a comprehensive nutrient data base enabled calculation of total daily nutrient intake and thus of consequences of milk exchange for dietary adequacy. Moreover, the background of a representative recent market survey in Germany made it easier to correctly classify the products that were selected for our calculations.

A limitation is that nutrient content of the selected products could not be confirmed by chemical analysis but had to be taken from the product labels.

### Conclusion

The fact that even a small reduction in nutrient-rich animal-based foods, such as replacing liquid milk by insufficient alternatives in a balanced children’s diet, has distinct effects on the micronutrient intake of young children poses major challenges for the proper information of parents. The assessment of even small interventions in a balanced diet need an expert view of advisors.

## Data Availability

No datasets were generated or analysed during the current study.
